# Antidote to Carbolic Acid

**Published:** 1872-06

**Authors:** 


### Antidote to Carbolic Acid.

A strong solution of saccharate of lime, it is asserted, is a reliable antidote against the poison of the carbolic acid, when by accident taken internally.

